# Evaluation of immunophenotypic markers and clinico-hematological profile in chronic lymphocytic leukemia: implications for prognosis

**DOI:** 10.1186/s13104-020-05243-7

**Published:** 2020-09-03

**Authors:** Marziye Bagheri, Tina Vosoughi, Mehran Hosseinzadeh, Najmaldin Saki

**Affiliations:** 1 Thalassemia and Hemoglobinopathy Research Center, Health Research Institute, Ahvaz Jundishapur University of Medical Sciences, Ahvaz, Iran; 2 Department of Laboratory Sciences, School of Allied Medical Sciences, Ahvaz Jundishapur University of Medical Sciences, Ahvaz, Iran

**Keywords:** Chronic lymphocytic leukemia, Immunophenotypic markers, Clinical parameters, Blood cell count, Prognosis

## Abstract

**Objective:**

Chronic lymphocytic leukemia (CLL) is an adult leukemia presented with clonal accumulation of lymphocytes. Immunophenotypic changes can be effective in predicting clinical course, the survival of patients, and determining first-line treatment. This is a study of the association between immunophenotypic markers with complete blood cell count (CBC) values and clinical parameters.

**Results:**

Peripheral blood samples were collected from 35 newly diagnosed CLL patients. The expression of immunophenotypic markers and CBC were evaluated. Platelet counts and hemoglobin concentration had a significant, inverse association with Rai staging, modified Rai staging, Binet staging systems (all p < 0.001 in both parameters), and splenomegaly (p = 0.001 and 0.007, respectively). The platelet/lymphocyte ratio (PLR) had a significant, inverse association with Rai staging (p = 0.014), modified Rai staging (p = 0.024), Binet staging systems (p = 0.027), and splenomegaly (p = 0.033). However, CD38, CD25, and double-positive CD56/CD117 expression, group 3 of innate lymphocyte cells (ILC3s), had no significant association with clinical parameters. In regression analysis, that ILC3s has an inverse correlation with neutrophil/lymphocyte ratio (r = −0.340, p = 0.046). Given that there is an inverse association between PLR and advanced clinical stages, it seems that PLR may have prognostic value in CLL.

## Highlights

- The platelet/lymphocyte ratio could be a prognostic marker which has correlation with an advanced clinical course in CLL.
- Lower platelet counts and Hb concentration association with advanced clinical course in CLL.
- There is an inverse correlation between innate lymphocyte cells and neutrophil/lymphocyte ratio in CLL.

## Introduction

Chronic lymphocytic leukemia (CLL) is one of the most common types of adult leukemia. It presents with the abnormal clonal accumulation of B lymphocytes in peripheral blood (PB), bone marrow (BM), or tissues [1–3].

CLL is categorized into prognostic groups based on the Rai and Binet clinical staging systems. In the Rai staging system, patients are classified into one of the five stages, stage 0 to IV, based on the presence or absence of lymphadenopathy, organomegaly, anemia, and thrombocytopenia [4, 5]. In the Binet staging system, patients are classified into three categories, stages A, B or C, based on the number of lymphoid tissues and the presence of anemia and/or thrombocytopenia [4–6].

Cytogenetic and molecular characteristics of CLL cells can be helpful in predicting clinical course, the survival of patients and determining the first-line treatment [4, 7]. Approximately 80% of CLL patients are detected by at least one cytogenetic abnormality. Deletions of the long arm of chromosome 13 (del 13q) and chromosome 11 (del 11q), the short arm of chromosome 17 (del 17p), and trisomy 12 are common chromosomal abnormalities which have prognostic values in CLL. It has shown that del 17p, del 11q, and trisomy 12 are associated with poor clinical outcomes such as high expression of ZAP-70 and CD38, immunoglobulin heavy chain variable gene (IGHV) unmutated, poor response to treatment, and shorter survival time and considered as adverse prognostic factors [5, 8].

CLL cells are characterized by high expression of CD5, CD19, and CD23 [2]. It seems that other markers, such as CD38, CD25, CD56, and CD117 may also have prognostic importance in CLL. As interleukin-2 (IL-2) receptor alpha chain, CD25 can be expressed in 30–50% of CLL patients. [1, 9]. CD38 participates in many cellular activities, including cell adhesion, and signal transduction. Some studies have shown that the expression of CD38 in CLL cells was associated with resistance to treatment, shorter survival, and aggressive clinical outcomes [1, 10] and high expression of CD38 can act as an adverse prognostic factor [11].

Innate lymphoid cells (ILCs), that comprise of three groups (ILC1s, ILC2s, and ILC3s), a family member of mononuclear hematopoietic cells, could play a key role in CLL pathogenesis by regulating adaptive immunity. ILC3s are characterized by CRTH22 (CD294)^−^ cKit (CD117)^+^ CD56^+/−^ and divided into natural cytotoxicity receptor (NCR)^+^ (NKp44 and NKp46) ILC3s and NCR^−^ ILC3s [12, 13].

Given the prognostic role of immunophenotypic markers in CLL patients, the aim of study was to assess the association between expression of CD38, CD25, and double-positive CD56 and CD117 (ILC3s) markers with peripheral complete blood cell count (CBC) values in CLL patients, and staging as defined by different schemes and clinical parameters.

## Main text

### Materials and methods

#### Patients and samples

We conducted a cross-sectional study confirmed from November 22, 2018 to September 21, 2019. The PB samples of 35 new CLL patients were collected. Patients were diagnosed according to the International CLL Workshop Criteria [14] and staged according to the modified Rai system [15]; patients were selected based on the following criteria: persistent lymphocytosis of more than 5.0 × 10^9^/L, cell morphology according to French American British (FAB) criteria, clinical features, and atypical immunophenotype with CD19+, CD20+, CD5+, CD23+, Ig light chain (kappa and lambda) as revealed by flow cytometry. Patient staging was done considering Binet or Rai clinical staging systems was based on physical examination and the size of liver and spleen.

#### Hematological analysis

Three mL of PB from each participant was collected in ethylenediamine tetra-acetic acid tubes and these samples were processed within 6–24 h of collection. CBC was performed by the automated hematology analyzer. Having an important role in CLL diagnosis and progress, several parameters of CBC were selected, including white blood cell (WBC), platelet count, hemoglobin (Hb), absolute lymphocyte counts (ALC) and, absolute neutrophil counts (ANC).

#### Flow cytometric analysis

Patients’ PB samples were analyzed using three-color flow cytometry. The expressions of immunophenotypic markers in patients shown in Additional file 1: Fig. S1a and Additional file 2: Fig. S1b. Directly labeled mouse MoAb were used (Dako, Denmark) against the lymphoid antigens CD45-peridinin chlorophyll protein (PerCP), CD5, CD25, and CD56-phycoerythrin (PE), CD19, CD20, CD23, and CD38-fluorescein isothiocyanate (FITC), and CD117-allophycocyanin (APC). The markers expression was analyzed by Partec Flow Cytometer (Partec PAS, Germany), and data were analyzed by FlowMax software.

#### Statistical analysis

All statistical analyses were performed using SPSS version 22, and data were expressed as median or interquartile range (IQR) for variables deviating from the normal distribution. Also, qualitative data were expressed as frequency and percentage. Normality was assessed by Kolmogorov–Smirnov test. We used the Mann–Whitney-U and Kruskal–Wallis tests to investigate the comparison of immunophenotypic markers or CBC results with clinical parameters. Spearman’s rho was used to study the correlation between nonparametric data. The level of significance was set at p < 0.05.

### Results

Thirty-five CLL patients including 24 males and 11 females, with a ratio of 2.1:1 (male/female) and the mean age of 64.34 years participated in this study. Results showed that (60.0%) of patients were at high-risk group, (17.1%) were at intermediate-risk group, and (22.9%) were at low-risk group. Almost half of the enrolled patients presented with splenomegaly (54.3%) and lymphadenopathy (31.4%). Regarding CBC results, (57.1%) of patients had anemia and (31.4%) thrombocytopenia. Clinical parameters, CBC and immunophenotypic markers of patients are summarized in Table 1.

**Table 1:** Clinical parameters, CBC and immunophenotypic markers of CLL patients

Characteristics	CLL patients (n = 35)
Age, mean (range)	64.34 (36–86 years)
Gender, n (%)	
Male	24 (68.6%)
Female	11 (31.4%)
Rai staging system, n (%)	
0	8 (22.9%)
I	3 (8.6%)
II	3 (8.6%)
III	10 (28.6%)
IV	11 (31.4%)
Modified Rai staging system, n (%)	
Low-risk group	8 (22.9%)
Intermediate-risk group	6 (17.1%)
High-risk group	21 (60.0%)
Binet staging system, n (%)	
A	12 (34.3%)
B	2 (5.7%)
C	21 (60.0%)
Splenomegaly, n (%)	
Yes	19 (54.3%)
No	16 (45.7%)
Lymphadenopathy, n (%)	
Yes	11 (31.4%)
No	24 (68.6%)
Hepatomegaly, n (%)	
Yes	0 (0%)
No	35 (100%)
WBC count × 10^3^/μL, median (IQR)	45 (22–112)
Platelets × 10^3^/μL, median (IQR)	131 (95–167)
≥ 100	n, 24 (68.6%)
< 100	n, 11 (31.4%)
Hemoglobin g/dL, median (IQR)	10.7 (9.9–13.2)
≥ 11	n, 15 (42.9%)
< 11	n, 20 (57.1%)
ALC × 10^3^/μL, median (IQR)	35.9 (18.4–101)
ANC × 10^3^/μL, median (IQR)	4.2 (3.6–11)
PLR × 10^3^/μL, median (IQR)	3.2 (1.3–8.1)
NLR × 10^3^/μL, median (IQR)	0.15 (0.09–0.18)
CD38 (%), median (IQR)	3.0 (1.0–6.0)
CD25 (%), median (IQR)	6.0 (1.5–10.0)
Double positive CD56/CD117, median (IQR)	0.1 (0.1–0.4)

*CLL* chronic lymphocytic leukemia, *WBC* white blood cell, *IQR* interquartile range, *ALC* absolute lymphocyte count, *ANC* absolute neutrophil count, *PLR* platelet/lymphocyte ratio, *NLR* neutrophil/lymphocyte ratio

#### Comparison of immunophenotypic markers with clinical parameters and CBC

The expression of immunophenotypic markers and CBC parameters were compared with clinical parameters (Table 2). It was apparent that there was no significant association between immunophenotypic markers (CD38, CD25, and double-positive CD56/CD117 expression) and each clinical parameter (lymphadenopathy, splenomegaly, Rai, modified Rai, and Binet staging systems). In the comparison of CBC results with clinical parameters of patients, it was shown that platelet count and Hb concentration had a significant association with Rai staging, modified Rai staging, Binet staging systems (all p < 0.001 in both parameters), and splenomegaly (p = 0.001 and 0.007, respectively). Also, there was a significant association between platelet/lymphocyte ratio (PLR) and Rai staging (p = 0.014), modified Rai staging (p = 0.024), Binet staging systems (p = 0.027), and splenomegaly (p = 0.033). In fact, platelet count, Hb concentration, and PLR were lower in patients in Rai stage IV, Binet stage C, high risk group or with splenomegaly. However, these parameters had no significant association with lymphadenopathy. In addition, no significant association were found in WBC count, ALC, ANC, and neutrophil/lymphocyte ratio (NLR) compared with clinical parameters.

**Table 2:** Comparison of the immunophenotypic markers expression and CBC with clinical parameters in CLL patients

Data	p value				
	Rai stage	Modified Rai stage	Binet stage	Splenomegaly	Lymphadenopathy
CD38	0.618	0.648	0.108	0.507	0.109
CD25	0.610	0.859	0.874	0.345	0.466
Double-positive CD56/CD117	0.573	0.650	0.558	0.211	0.854
WBC count × 10^3^/μL	0.239	0.274	0.272	0.312	0.195
Platelets × 10^3^/μL	< 0.001**	< 0.001**	< 0.001**	0.001**	0.606
Hemoglobin g/dL	< 0.001**	< 0.001**	< 0.001**	0.007**	0.709
ALC × 10^3^/μL	0.241	0.273	0.261	0.289	0.201
ANC × 10^3^/μL	0.254	0.535	0.907	0.145	0.749
PLR × 10^3^/μL	0.014*	0.024*	0.027*	0.033*	0.311
NLR × 10^3^/μL	0.292	0.115	0.147	0.327	0.354

*CLL* chronic lymphocytic leukemia, *WBC* white blood cell, *ALC* absolute lymphocyte count, *ANC* absolute neutrophil count, *PLR* platelet/lymphocyte ratio, *NLR* neutrophil/lymphocyte ratio

*Indicates statistical significance (p < 0.05)

**Indicates statistical significance (p < 0.01)

#### Correlation of immunophenotypic markers with CBC

The results of the regression analysis showed that double-positive CD56/CD117 expression had a significant, inverse correlation with NLR (r = −0.340, p = 0.046) (Additional file 3: Fig. S2), while expression of double-positive CD56/CD117 had non-significant, inverse correlation with PLR (Additional file 4: Fig. S3). The expression of CD38 and CD25 had no significant correlation with WBC count, platelet count, Hb concentration, ALC, ANC, PLR, and NLR (Table 3). Although CD38 expression had no significant correlation with NLR (r = 0.307, p = 0.073), the higher percentage of this marker was associated with higher NLR. However, the results indicate that WBC count had a direct correlation with ALC (r = 0.995, p < 0.001), but an inverse correlation with Hb concentration (r = −0.349, p = 0.040), PLR (r = −0.920, p < 0.001), and NLR (r = −0.527, p = 0.001). Also, the ALC had an inverse correlation with Hb concentration (r = −0.355, p = 0.036), PLR (r = −0.914, p < 0.001), and NLR (r = −0.589, p = 0.001). There was a direct correlation between Hb concentration and platelet count (r = 0.691, p < 0.001), PLR (r = 0.535, p = 0.001), and NLR (r = 0.365, p = 0.031).

**Table 3:** Correlation between immunophenotypic markers expression with CBC in CLL patients

Parameters	CD38%		CD25%		Double-positive CD56/CD117%	
	r	p	r	p	r	p
Age	− 0.075	0.669	− 0.233	0.179	0.005	0.978
WBC count × 10^3^/μL	− 0.207	0.233	0.196	0.258	0.251	0.146
Platelets × 10^3^/μL	0.096	0.582	− 0.171	0.326	− 0.178	0.307
Hemoglobin g/dL	0.047	0.788	− 0.135	0.440	− 0.167	0.337
ALC × 10^3^/μL	− 0.218	0.207	0.175	0.315	0.275	0.110
ANC × 10^3^/μL	− 0.063	0.718	0.243	0.160	0.223	0.198
PLR × 10^3^/μL	0.163	0.349	− 0.199	0.252	− 0.264	0.125
NLR × 10^3^/μL	0.307	0.073	0.020	0.908	− 0.340	0.046*

*CLL* chronic lymphocytic leukemia, *WBC* white blood cell, *ALC* absolute lymphocyte count, *ANC* absolute neutrophil count, *PLR* platelet/lymphocyte ratio; *NLR* neutrophil/lymphocyte ratio; *p* p value; *r* regression

*Indicates statistical significance (p < 0.05)

### Discussion

It seems that assessing clinical course of the CLL disease combined with molecular and biological factors can predict disease progression and patient’s survival [1, 5]. In this regard, we investigated the changes in the expressions of different immunophenotypic markers in CLL patients to explore prognostic value of these markers.

There was no significant association between CD38 expression and each clinical parameter (lymphadenopathy, splenomegaly, Rai staging, modified Rai staging, and Binet staging systems). This finding is consistent with that of Abdelgader et al. and Kamel et al. studies, which reported no significant association between CD38 expression and clinical parameters [16, 17]. In contrast, Ibrahim et al. showed that CD38 expression is associated with hepatomegaly and aggressive clinical stages [9]. Also, the significant association between CD38 expression and splenomegaly, intermediate and high-risk disease was reported by some other studies [18, 19]. In contrast to some reports, we found that the percentage of CD38 expression has no significant correlation with CBC results. Studies have reported that CD38 expression has a direct correlation with WBC and lymphocyte count [20, 21] and an inverse correlation with Hb concentration and platelet count [10, 16, 18, 20]. Although there was not any significant correlation between strength of CD38 expression and NLR (r = 0.307, p = 0.073), the higher percentage of this marker was associated with higher NLR. Given the direct correlation between CD38 expression, as an adverse prognostic factor, and NLR in CLL, further research is warranted to investigate whether NLR can be considered as a poor prognostic factor in this disease.

Another finding was that CD25 expression had no significant association with clinical parameters. These results differ from Shvidel et al. and Grywalska et al. studies, it was reported that CD25 expression had a significant association with splenomegaly and Rai stage III [21, 22]. Unlike Hjalmar et al. study, indicated that CD25 expression associated with lymphocyte count [9], we did not find any significant correlation between CD25 expression and CBC results.

Our results did not show any significant association between ILC3s and clinical parameters (lymphadenopathy, splenomegaly, Rai staging, modified Rai staging, and Binet staging systems), although we found an inverse correlation between these cells and NLR. Weerdt et al. in a similar study showed that ILCs count significantly increased in CLL patients and had a direct correlation with ALC and an inverse correlation with time to first treatment [23]. It seems that future investigations might be able to reveal the exact role of ILCs in CLL pathobiology.

Here, it was shown that platelet count, Hb concentration, and PLR are significantly associated with Rai staging, modified Rai staging, Binet staging systems, and splenomegaly. So that, the platelet count, Hb concentration, and PLR were lower in patients in Rai stage IV, Binet stage C, high risk group or with splenomegaly. Similarly, Basabaeen et al. showed that thrombocytopenia was significantly correlated with splenomegaly [24]. However, Sall et al. study reported that lymphocyte count was significantly greater in patients with stage C than groups with stages A or B [25]. Furthermore, Ahmed et al. in a study reported that there was not significant associated between CBC results and advanced Rai stage [26]. Although Bakouny et al. did not find significant relation between PLR with the survival of CLL patients [27], there was an inverse correlation between PLR and advanced clinical stages of CLL in our study. Considering the overall findings, we can hypothesis that PLR can act as a prognostic biomarker in CLL patients which requires further investigation.

The aim of study was to determine the prognostic role of immunophenotypic markers of CLL patients and their relevance to clinical parameters and hematological parameters. PLR had an inverse association with patients’ outcome, suggesting that it may have a prognostic value in CLL patients which requires further investigation. Moreover, we found that ILC3s had an inverse correlation with NLR, but it was difficult to arrive at any conclusion with regard to the prognostic role of these cells. Thus, further studies are required to determine the prognostic role of these cells in CLL patients.

## Limitations

- Patients were not followed up for the progression of CLL, survival rates and response to treatment.
- The prognostic role of cytogenetic findings of patients was not evaluated.
- The cytokine production as a marker for ILCs functionality was not evaluated.

## Supplementary information

**Additional file 1: Fig. S1.** Representative flow cytometry profiles of immunophenotypic markers expression in patients with CLL. a) Sample of CD38 and CD25 expression.

**Additional file 2: Fig. S1.** Representative flow cytometry profiles of immunophenotypic markers expression in patients with CLL. b) Sample of double-positive CD56/CD117 expression.

**Additional file 3: Fig. S2.** A significant, inverse correlation between double-positive CD56/CD117 expression with neutrophil/lymphocyte ration (NLR) (r = −0.340, p = 0.046) in CLL patients.

**Additional file 4: Fig. S3.** A non-significant, inverse correlation between double-positive CD56/CD117 expression with platelet/lymphocyte ration (PLR) (r = −0.264, p = 0.125) in CLL patients.

## Data Availability

The datasets used analyzed during the current study are available from the corresponding author on reasonable request.

## Abbreviations

CLL: Chronic lymphocytic leukemia
PB: Peripheral blood
BM: Bone marrow
ILCs: Innate lymphoid cells
CBC: Complete blood cell count
WBC: White blood cell
Hb: Hemoglobin
ALC: Absolute lymphocyte counts
ANC: Absolute neutrophil counts
PLR: Platelet/lymphocyte ratio
NLR: Neutrophil/lymphocyte ratio
