# Supplementary figures and images for: Evaluation of immunophenotypic markers and clinico-hematological profile in chronic lymphocytic leukemia: implications for prognosis
Source: BMC Res Notes. 2020 Sep 3;13:412. doi: 10.1186/s13104-020-05243-7 (PMC7469386; doi:10.1186/s13104-020-05243-7)

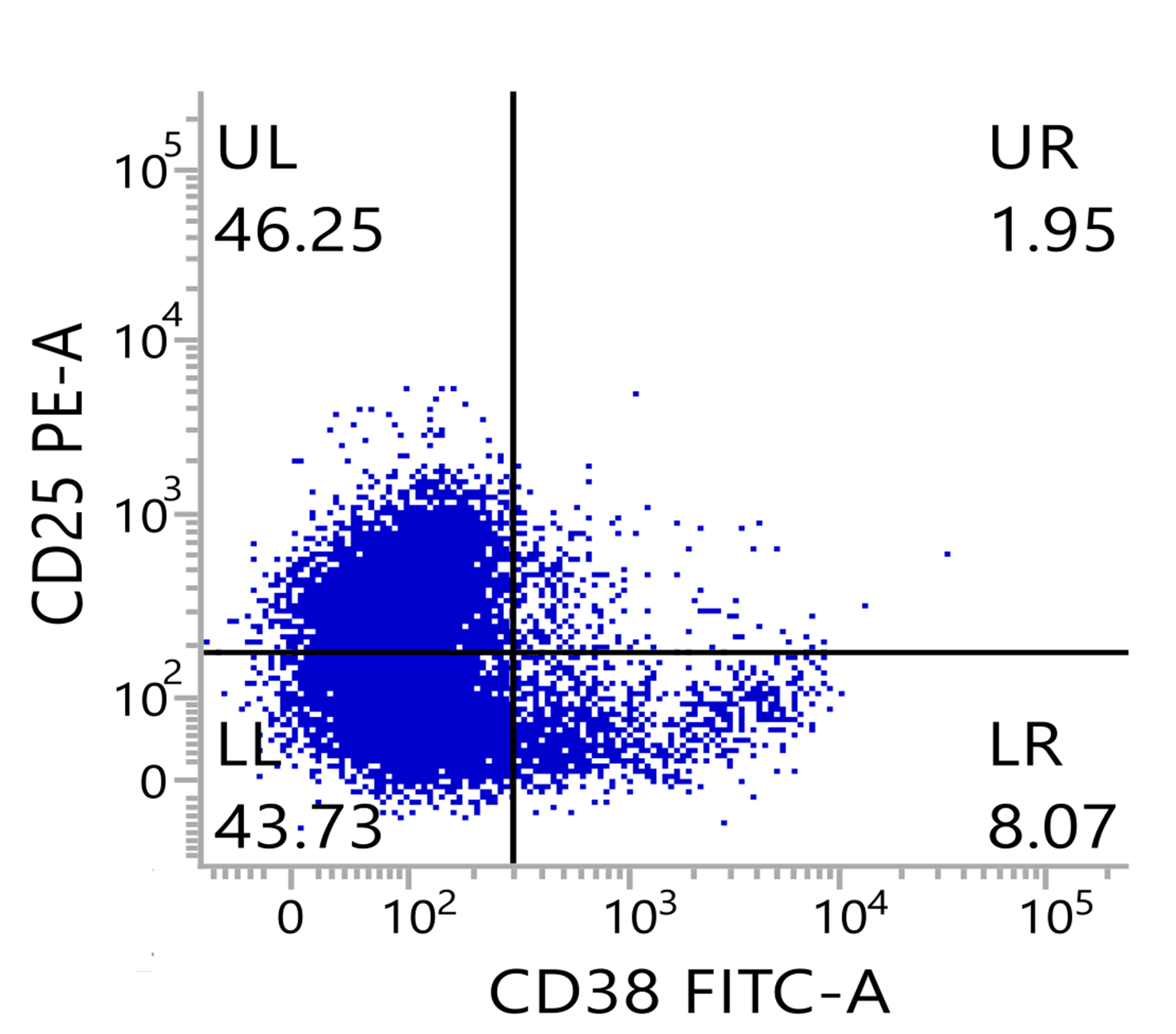

Supplement: Supplementary file 1 — Additional file 1: Fig. S1. Representative flow cytometry profiles of immunophenotypic markers expression in patients with CLL. a) Sample of CD38 and CD25 expression. [file 13104_2020_5243_MOESM1_ESM.tif]

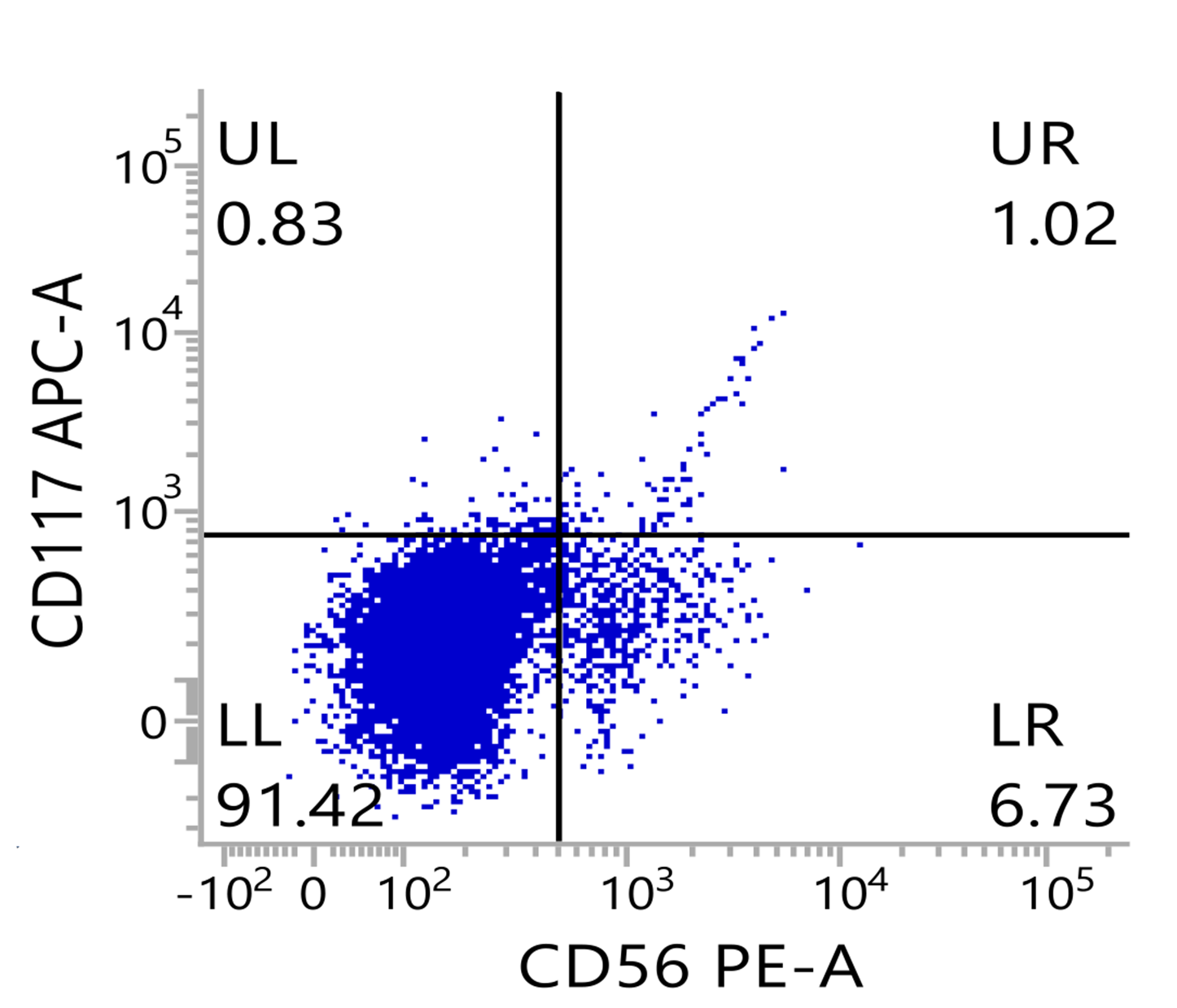

Supplement: Supplementary file 2 — Additional file 2: Fig. S1. Representative flow cytometry profiles of immunophenotypic markers expression in patients with CLL. b) Sample of double-positive CD56/CD117 expression. [file 13104_2020_5243_MOESM2_ESM.tif]

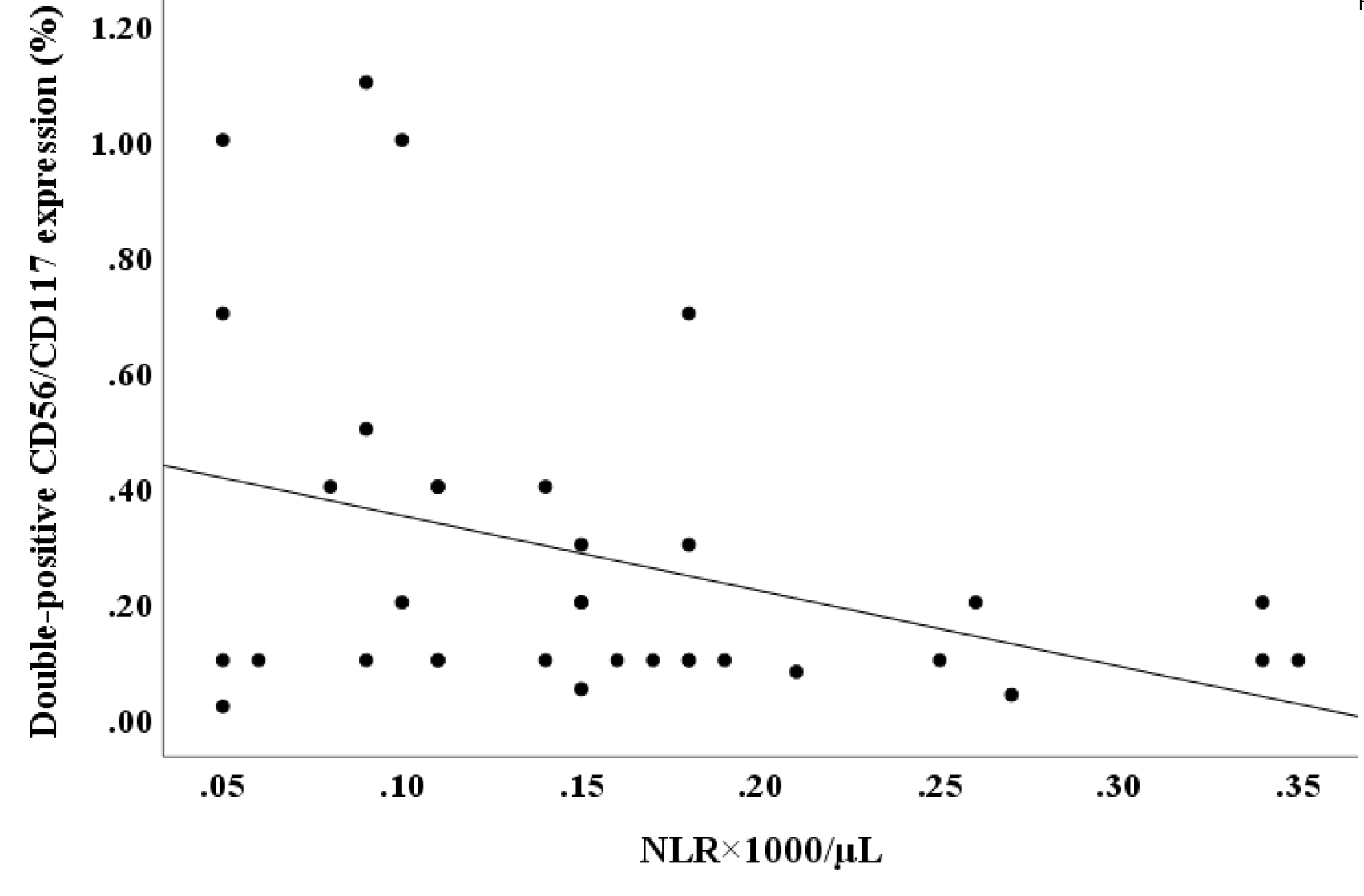

Supplement: Supplementary file 3 — Additional file 3: Fig. S2. A significant, inverse correlation between double-positive CD56/CD117 expression with neutrophil/lymphocyte ration (NLR) (r = −0.340, p = 0.046) in CLL patients. [file 13104_2020_5243_MOESM3_ESM.tif]

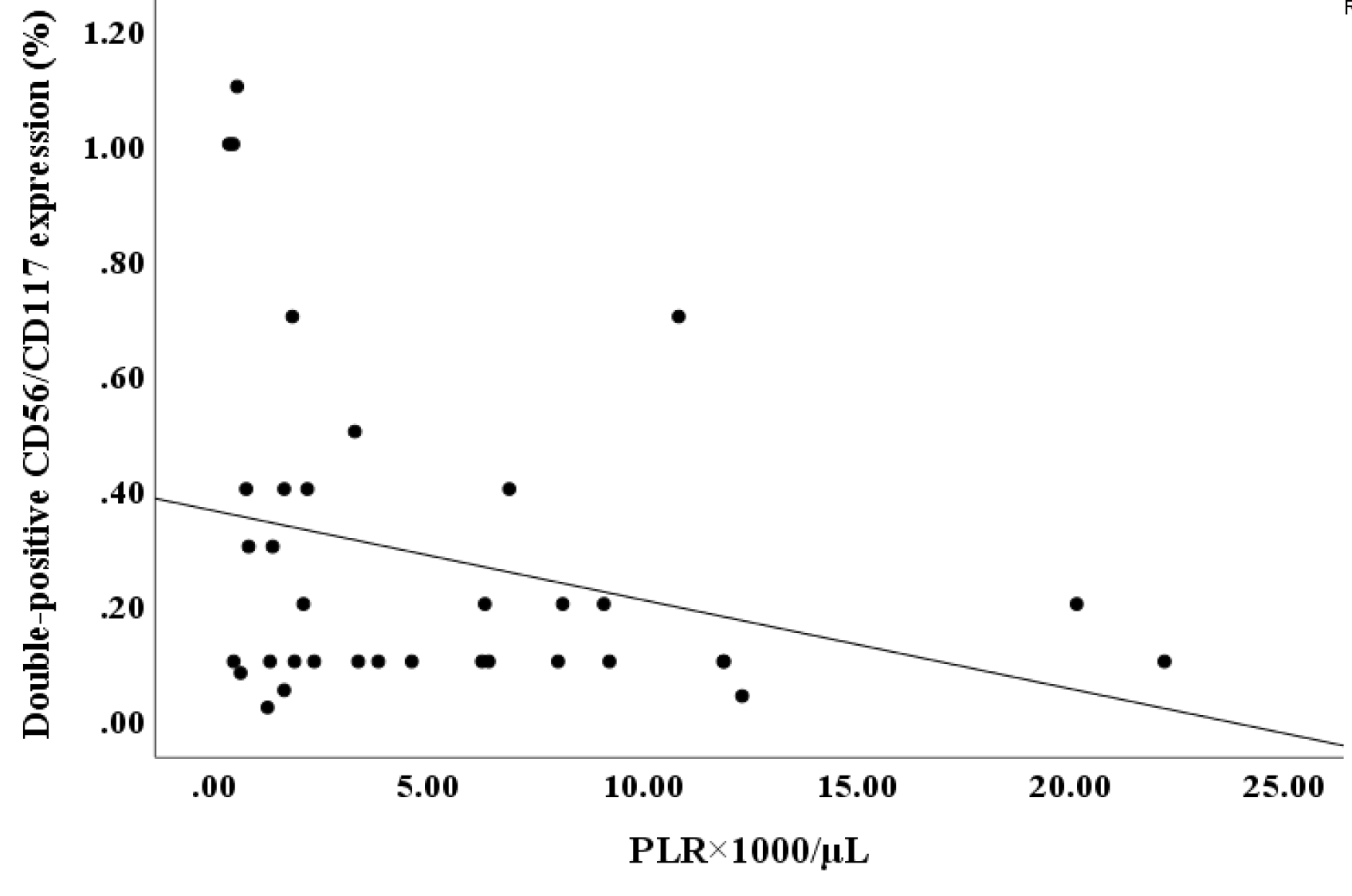

Supplement: Supplementary file 4 — Additional file 4: Fig. S3. A non-significant, inverse correlation between double-positive CD56/CD117 expression with platelet/lymphocyte ration (PLR) (r = −0.264, p = 0.125) in CLL patients. [file 13104_2020_5243_MOESM4_ESM.tif]
